# The Involvement of Krüppel-like Factors in Cardiovascular Diseases

**DOI:** 10.3390/life13020420

**Published:** 2023-02-02

**Authors:** Michelle G. Santoyo-Suarez, Jimena D. Mares-Montemayor, Gerardo R. Padilla-Rivas, Juan Luis Delgado-Gallegos, Adriana G. Quiroz-Reyes, Jorge A. Roacho-Perez, Diego F. Benitez-Chao, Lourdes Garza-Ocañas, Gilberto Arevalo-Martinez, Elsa N. Garza-Treviño, Jose Francisco Islas

**Affiliations:** 1 Departamento de Bioquímica y Medicina Molecular, Universidad Autónoma de Nuevo León, Monterrey 64460, Mexico; 2 Departamente de Farmacología y Toxicología, Facultad de Medicina, Universidad Autónoma de Nuevo León, Monterrey 64460, Mexico

**Keywords:** cardiovascular diseases, Krüppel-like factors, transcription factor regulation

## Abstract

Krüppel-like factors (KLFs) are a set of DNA-binding proteins belonging to a family of zinc-finger transcription factors, which have been associated with many biological processes related to the activation or repression of genes, inducing cell growth, differentiation, and death, and the development and maintenance of tissues. In response to metabolic alterations caused by disease and stress, the heart will undergo cardiac remodeling, leading to cardiovascular diseases (CVDs). KLFs are among the transcriptional factors that take control of many physiological and, in this case, pathophysiological processes of CVD. KLFs seem to be associated with congenital heart disease-linked syndromes, malformations because of autosomal diseases, mutations that relate to protein instability, and/or loss of functions such as atheroprotective activities. Ischemic damage also relates to KLF dysregulation because of the differentiation of cardiac myofibroblasts or a modified fatty acid oxidation related to the formation of a dilated cardiomyopathy, myocardial infarctions, left ventricular hypertrophy, and diabetic cardiomyopathies. In this review, we describe the importance of KLFs in cardiovascular diseases such as atherosclerosis, myocardial infarction, left ventricle hypertrophy, stroke, diabetic cardiomyopathy, and congenital heart diseases. We further discuss microRNAs that have been involved in certain regulatory loops of KLFs as they may act as critical in CVDs.

## 1. Introduction

In response to metabolic alterations caused by disease and stress, the heart undergoes changes that are referred to as pathological remodeling, which involves hypertrophy and fibrosis, eventually leading to cardiac failure [1,2]. Similar metabolic changes can also affect the blood vasculature, which leads to structural alterations that potentially evolve into angiogenesis and atherosclerosis. These affections of heart and blood vessels are termed cardiovascular diseases (CVDs) [3,4,5]. To date, CVDs remain as the leading cause of mortality worldwide, claiming up to 18.56 million lives just in 2019, according to data of the Global Burden of Disease Study [6]. For the United States (U.S.), CVDs and stroke continue to be in the top 10 causes of death according to 2021 reports, with 173.8 and 41.1 deaths per 100,000 U.S. habitants, respectively [7]. Additionally, some of the most prevalent risk factors associated with premature death, such as high blood pressure, smoking, high blood sugar, and obesity, are well-known to be associated with the development of CVDs [6].

Cardiovascular diseases have a multifactorial etiology, which has not been fully clarified up to now. In recent years, the family of Krüppel-like transcription factors (KLFs) have acquired traction, as new developments have shed light on their involvement in various processes, including those associated with CVDs. The KLFs were first identified and characterized in the early 1990s as erythroid cell-specific transcription factors [8], yet since then, several KLFs have been isolated in plenty of organs and tissues. Krüppel-like factors are a set of DNA-binding proteins belonging to the family of zinc-finger transcription factors [9]. Krüppel-like factors were named after their similarity to the *Drosophila melanogaster* Krüppel protein (from German, “crippled” protein), a member of the *gap* gene class involved in the thorax and anterior abdomen segmentation of *Drosophila* embryos [10], which, when presenting alterations, result in severe body abnormalities [11]. Krüppel-like factors are important regulators of gene transcription and can activate or repress the expression of genes involved in a variety of processes, including cell growth, differentiation, and death, and the development and maintenance of specialized tissues, under both physiological and pathological conditions [12]. Over the past few decades, numerous studies have explored the role of KLFs in CVDs, revealing that KLFs are involved in the regulation of a wide range of processes that are relevant to CVDs, including inflammation, oxidative stress, and cell proliferation [13,14,15,16]. For instance, KLF-2 has been associated with endothelial activation, possessing an anti-inflammatory effect and thus, being protective against CVDs [13]. On the other hand, KLF-5 has been found to be pro-inflammatory and pro-proliferative and has been implicated in the progression of CVDs [17].

However, much remains to be learned about the precise mechanisms by which KLFs contribute to CVDs and about the potential of these proteins as therapeutic targets for the treatment of these diseases. As such, research on KLFs and their roles in CVDs is ongoing and is likely to continue in the future. The aim of this review is to summarize what is known until now regarding CVDs, including atherosclerosis, ischemic diseases, myocardial infarction, stroke peripheral artery diseases, and congenital heart disease. Additional research has also linked the involvement of microRNAs as regulators of KLFs, hence we further devote a section to this topic. Our work was accomplished through comprehensive bibliographic research with the selection of the most relevant and recent findings in the topics.

## 2. Krüppel-like Factors Structure and Domains

To date, researchers have identified 17 KLFs in the human genome, excluding the putative *KLF-18* gene, which arises as a duplication of *KLF17* [18]. Krüppel-like factors are characterized by their three well-conserved carboxyl terminal C2H2 zinc-finger domains, where two cysteines coordinate each zinc ion at one end of a β-sheet, and two histidines at the C-terminal α-helix, creating a tetrahedral structure that allows for folding ββα protein configuration [19]. These zinc fingers are highly conserved throughout the family, sticking to the consensus sequence C-X2-5-C-X3-(F/Y)-X5-ψ-X2-H-X3-5-H, where X represents any amino acid and ψ is a hydrophobic residue [20]. A seven amino acid spacer TGEKP(Y/F)X can be found between each finger [21]. The first two zinc fingers have 25 amino acids each, while the third has only 23; at a particular level, each zinc finger can bound three base pairs in GC-rich regions, such as CACCC-, GC-, and GT-box elements, located in promoters of target genes [22]. The amino-terminal domain is extremely varied and arranged, containing binding domains that are capable of exerting repressive or activating functions. Finally, a nuclear localization signal (NLS) can be found near or within zinc fingers [23].

### Krüppel-like Factors Phylogenetic Classification

Research by McConnell et al. (2010) established a consensus to subdivide KLFs into three phylogenetic groups and one non consensus group in relation to their structural similarities and binding domains (Table 1).

**Table 1:** Krüppel-like factors phylogenetic groups.

Group Members	Description	References
Group 1 KLF-3 KLF-8 KLF-12	These mediate transcriptional repression by binding their C-terminal domain to the CtBP protein. CtBP can then mediate co-repression in an HDAC-dependent process, allowing histones to wrap DNA tightly. This mechanism was assessed by Turner and Crossley when they proved that mutations in the CtBP-binding motif in KLF-3 failed to repress gene expression in SL2 cells. A gene repression HDAC-independent process could be executed by CtBP recruitment of PcG-associated proteins complex.	[24,25,26,27]
Group 2 KLF-1 KLF-2 KLF-4 KLF-5 KLF-6 KLF-7	They mostly operate as transcriptional activators by recruiting acetyltransferase activity factors, such as CBP, p300, and P/CAF, promoting chromatin remodeling. Nevertheless, KLF-2 and KLF-4 also contain domains with repressor functions, which are continuous to the activation domains.	[28,29]
Group 3 KLF-9 KLF-10 KLF-11 KLF-13 KLF-14 KLF-16	They have mostly been described as transcriptional repressors through their binding to SinA3. This interaction is possible because of a hydrophobic consensus sequence in these KLFs N-terminal domains, a conserved α-helical motif AA/VXXL that mediates their linking to SinA3 paired amphipathic helix domain, which then works as a scaffold for other chromatin modifiers, such as HDAC1, HDAC2, Mad, Ume6, MeCP2, N-CoR, and Ikaros.	[28,30]
No consensus group. KLF-15KLF-17 (-18)	These factors have not been incorporated into any of these phylogenetic groups since their interaction domains remain undetermined. Yet, tissue expression in bone, kidney, and testis has been reported.	[11,18,31]

Krüppel-like factors as a family are found in several organ systems, namely the hematopoietic, gastrointestinal, respiratory, nervous, immune, and cardiovascular [32]. Hence, it is not uncommon for KLFs to have a ubiquitous expression pattern, such as in *KLFs 6*, *7*, *8*, *9*, *10*, and *11*. Others have a more restricted expression, such as *KLF-1*, which is present in erythroid and mast cells, and *KLF-2*, which is involved in lung and vessel development [23,33]. Both *KLF-1* and *KLF-2* play an essential role in embryonic erythropoiesis, since they can bind to genes involved in cell proliferation and cell cycle control, such as *Forkhead Box M1 (FoxM1)*, *Spinghosine kinase 1 (Sphk1)*, *Parathyroid hormone 1 receptor (Pthr)*, and CD24a antigen, thus promoting the maturation of erythroid precursors [33].

Regarding the heart, KLFs expression and function continue at the initial elucidation stages. *KLF-15* expresses in cardiomyocytes and cardiac fibroblasts and upregulates post-natally, inhibiting cardiac hypertrophy by preventing myocardin (MYOCD) and serum response factor (SRF) interactions, thus, diminishing *atrial natriuretic* factor (*ANF*) and *α-skeletal actin* (*α-SKA*) expression [34,35]. Meanwhile, *KLF-13* has a marked reduced expression during the post-natal stage; moreover, it also works as a cofactor with GATA4 and TBX5, an essential part of the transcriptional machinery required for inducing cardiac cell differentiation. Deletion of *KLF-13* (as well as other GATA4 modifier factors) has been associated with congenital heart defects, including Holt–Oram syndrome (discussed in a subsequent section), atrial and septal malformations, and ventricular hypotrabeculation [36].

## 3. Cardiovascular Diseases (CVDs)

Cardiovascular diseases continue to be at the top of the list of causes of death worldwide [37,38]. Cardiovascular diseases affect both the blood vessels, as well as the heart at the mechanical, electrical, and cellular level, directly compromising nutrition and oxygenation, leading to damage and eventually death of the affected region [39,40,41]. At a fundamental level, we should note that the heart has little room for regeneration; therefore, damaged cardiomyocytes or other cardiac cells will eventually lead to cell loss, fibrosis, and heart failure [42,43,44]. The term CVDs is quite broad, encompassing a wide spectrum of diseases such as ischemic heart disease, heart failure, valvular heart disease, arrhythmias, high blood pressure, stroke, and others [45]. Yet, the most common causes of morbidity and mortality associated with CVDs are ischemic heart disease, stroke, and heart failure, which account for nearly 80% of all CVDs globally [41,46,47,48].

Interestingly, much information about the risk factors involved in developing CVDs are known. Many of these risk factors are preventable by behavioral changes. These preventable risk factors include tobacco consumption, physical inactivity, obesity, and unhealthy eating habits [49]. As an example of the latter, research has shown that a high lipid intake in the diet is associated with the development of atherosclerotic plaques, a condition directly related to CVDs [50,51]. Researchers have further linked high fat consumption and dietary obesity to an induced state of inflammation, generating adipose tissue and increasing the secretion of pro-inflammatory cytokines such as NFκB, TNF-α, and INF-γ [52]. This inflammatory state induces the production of reactive oxygen species (ROS) in the mitochondria, which leads to lipid peroxidation that can eventually induce several pathologies, including Alzheimer’s and the development of aneurysms [53]. Excessive fatty acids lead to triglyceride and cholesterol esterification. Next, these lipids are taken up by VLDL and are later directed to LDL. In an already primed inflammatory state, high LDL levels can become oxidized (ox-LDL). Ox-LDL then becomes a problem, as signals lead macrophages to engulf them, becoming foam cells that stack up in the arteries over time, eventually forming atherosclerotic plates [54]. Another preventable source of high ROS levels and oxidation is tobacco consumption. Particularly, vascular smooth muscle cells (VSMCs) can react to external stimuli. Changes in these cells directly affect their differentiation from contractile cells to cells concerned with inflammation and ECM remodeling, reducing their expression of *alpha smooth muscle actin (α-SMA)* and *smooth muscle 22 alpha (SM22α)*, and enhancing the production of inflammatory mediators as previously described with an outcome of atherosclerosis progression [55]. Unfortunately, not all risk factors are preventable, such as hyperglycemia related to type-1 diabetes, which has a genetic component, and its prevention is more complex. In this situation, a healthy lifestyle continues to be paramount [56]. At a molecular level, cardiac regulation and function involve a plethora of transcriptional factors that specify genes that take control of many physiological and, in this case, pathophysiological processes of CVDs [57]. One subset family of transcriptions factors involved in cardiac regulation during pathophysiological processes of CVDs is the KLF family.

### 3.1. Krüppel-like Factors in Atherosclerosis

Atherosclerosis is a phenomenon characterized by the deposition of ox-LDL cholesterol in the arterial walls and is the primary pathology of CVD [54]. It develops from endothelial dysfunction, LDL retention, and the occupation of leukocytes in the subendothelial space, followed by the signaling, recruitment, and differentiation of macrophages. Through the induction of nitric oxide synthase (eNOS and iNOS) and LDL oxidation, macrophages transform into foam cells, eventually forming atherosclerotic plates [58]. This change favors the evolution toward fibrous plaques that progressively reduce the diameter of the arterial lumen. Turbulent blood flow through a partially occluded vessel (or even normal fluctuations in the blood vessel path, such as in arterial bifurcations) damages the endothelium by shear force [58,59].

At the molecular level, *KLF-2* activation has been associated with laminar blood flow, a key protective force in the arterial walls that helps prevent atherosclerosis, since it induces a protective phenotype in endothelial cells. In low-shear stress regions, KLF-2 inhibits a mechanosensory complex composed of platelet endothelial cell adhesion molecule (PECAM-1), vascular endothelial cadherin (VE-cadherin), and vascular endothelial growth factor receptor 2/3 (VEGFR2/3). These factors trigger the MEK2/ERK2 pathway to upregulate myocyte enhancer factor-2 (MEF2) and allow *KLF-2* transcriptional activity. KLF-2 exerts, as it downregulates vascular adhesion molecule-1 (VCAM-1) and E-selectin, molecules that support leukocyte migration and adhesion [60]. Researchers have identified suberanilohydroxamic acid as a potent pharmacological inducer of KLF-2, capable of repressing vascular inflammation and atherosclerosis [61]. Researchers believe that the main mechanism for this repression is the inhibition of thrombin-mediated cytokine as a repression mechanism of the protease-activated receptor 1 (PAR-1) [14].

According to Xie et al. (2021), one of the key changes in the progression of atherosclerosis is the transition of VSMCs from a contractile phenotype to a proliferative phenotype. This transition leads to an increase in extracellular matrix secretion, resulting in the formation of arterial intima layer thickening. In this regard, the *KLF-5* expression evidence seems to indicate an elevation in atherosclerotic plaques compared to normal human aortic tissue, suggesting that *KLF-5* may play a role in promoting this phenotypic switch [62].

Transiently induced *KLF-4* after a vascular injury is not constitutively expressed in VSMCs [63]. In animal models, researchers found that after carotid artery ligation, KLF-4 activates rapidly in the VSMC, which inhibited the expression of VSMC differentiation marker genes (*SM-22* and *α-SMA*), as the evidence suggests that KLF-4 blocks these markers through the binding of *TGF-*βs control element-containing promoter (5′-GAGTGGGGCG-3′). In contrast, no binding of KLF-4 has been shown in intact carotid arteries [64,65,66]. Moreover, *KLF-4 KO* mice exhibited enhanced neointimal proliferation after vascular injury, contributing to the reduced arterial lumen. These results suggest that *KLF-4* is a negative regulator of neointima formation [67]; furthermore, the effects on non-vascular endothelial cells have also been observed. KLF-4 does not affect SMC differentiation markers, but it downregulates *TNF-α*-induced *VCAM1* expression by targeting and blocking the binding site of NFκB to the *VCAM1* promoter. Adhesion molecule expression promotes the accumulation of inflammatory cells that contribute to neointima formation [16].

Atherosclerosis and the shear stress forces associated with it led to plaque rupture, causing thrombosis or vascular embolism, giving rise to ischemic heart disease in any of its two main clinical forms: angina or acute myocardial infarction (AMI) [68].

### 3.2. Krüppel-like Factors in Ischemic Disease, Remodeling, and Heart Failure

Myocardial infarction (MI) is the most severe clinical manifestation of ischemic heart disease. It comprises the abrupt obstruction of blood flow in the main branches of the coronary arteries, eventually leading to cardiomyocyte ischemia [4,38,41]. In ischemic heart disease progression, fibrotic tissue replaces damaged muscle inducing geometric, biomechanical, and biochemical changes in the heart. This process is crucial to prevent ventricular wall rupture in the post-infarction period; however, an exaggerated fibrotic response has detrimental effects, leading to heart remodeling and a progressive loss of cardiovascular function to establish heart failure since it does not restore blood flow [69]. There is an affection of the myocardial tissue that leads to an overall decrease in oxygen or hypoxia, which ultimately causes necrosis of the area. Interestingly enough, as hypoxia and stress increases, there is an overall shift in signaling, which renders the activation of a fetal program in the tissue, which is characteristic of development [70]. Elevation of embryonic signaling, as was seen in eight patients treated with pressure-controlled intermittent coronary sinus occlusion (PISCO), resulted in the elevation of the transcription factors GATA4, MEF2C, TBX5, and HAND2 in blood samples. These are particular cardiac transcription factors, which have a history of being used in direct differentiation studies [71,72,73]. In the PISCO study, patient serums were collected and co-cultured with human fibroblasts and cardiomyocytes. Their findings indicated an upregulation in KLF-4: a known pluripotent stem cell inducer [63,74,75,76]. Unsurprisingly, KLF-4 promotes cardiac myofibroblast differentiation and collagen synthesis in angiotensin II-induced cardiac fibrosis through its binding to the *TGF-β1* promoter, activating the TGF-β1/Smad3 pathway, increasing the expression *a-SMA* and the secretion of type I and type III collagen, and contributing to the induction of a proliferative phenotype in cardiomyocytes [77].

Previously identified KLF-5 is a prohypertrophic factor that is increased in patients with terminal heart failure and mice with ischemic cardiomyopathy. The exact mechanisms by which KLF-5 induces cardiac hypertrophy remain unknown; however, research by Hoffman et al. confirmed that in mice subjected to left coronary artery ligation, *Klf-5* expression increased 2-fold after 24 h and 4-fold after 2 and 4 weeks. A reduction in fractional shortening and expansion of the end-diastolic and systolic dimensions accompanied *Klf-5* upregulation. When using the pharmacological inhibitor of *Klf-5*, ML264, an improvement in echocardiographic parameters, such as ejection fraction, was observed, as well as a reduction in end-diastolic and systolic volume, exerting a protective effect against ischemic cardiomyopathy [78].

Previous research showed that KLF-5 could regulate *PPAR-α* expression and modify fatty acid oxidation (FAO). The heart depends on FAO to produce ≈70% of its ATP and meet its energy demands [79]. This process transcriptionally depends on *PPAR-α*, which KLF-5 can activate via direct promoter binding [15,47]. The cardiac myocyte-specific ablation of KLF-5 consequently resulted in a decrease in *PPAR-α*, FAO, cardiac ATP levels, and triacylglycerol accumulation [47]. Interestingly, even though KLF-5 was being suppressed, the experimental model indicated signs of dilated myocardiopathy, such as a reduction of fractional shortening and an increase in left ventricle internal dimensions, showing that an excessive accumulation of lipids in the heart can indeed lead to dilated cardiomyopathy [15,47]. Although in this study, cardiomyopathy was suggested to develop in a KLF-independent manner, recent evidence shows a link between KLF-5 and ceramide biosynthesis. KLF-5 has been proposed as a direct transcriptional regulator of *SPTLC1* and *SPTLC2* (serine palmitoyltransferase [SPT] long-chain base subunit 1 and 2, respectively), enzymes involved in the rate-limiting step of ceramides de novo pathway synthesis, producing ceramides from serine and palmitoyl coenzyme A [15,78].

Regarding diabetic cardiomyopathy (DbCM), KLF-5 has been linked to oxidative stress via the upregulation of NADPH oxidase 4 (NOX4) by directly binding to NADPH oxidase 4 promoter, inducing *NOX4* expression, and leading to cardiomyocyte superoxide accumulation, mitochondrial abundance decrement, and a change in the cardiac lipidome profile toward a ceramide-rich environment; therefore, contributing to DbCM physiopathology [15]. Meanwhile, dilated cardiomyopathy (DCM) is the most frequent cause of heart failure in young people. In the most severe cases, it is also a major reason for a heart transplant [80,81,82]. Krüppel-like factor-5 has recently been documented as being highly involved in the development of DCM. According to whole exome sequencing studies, *KLF-5* mutations were directly responsible for DCM with complete penetration within the proband’s family members [81]. Hence, KLF-5 inhibition has been proposed as a strategy to treat heart failure and other cardiovascular diseases [15,17].

Genetic variations of *KLF-15*, documented as a hypertrophy inhibitor, have been studied in patients with type 2 diabetes. These findings have shown that a single nucleotide variation (SNV) in intron two of the *KLF-15* gene (rs9838915) was associated with increased left ventricle mass index and septal wall thickness. An additional follow-up of 5.6 years on average was performed, where 22 patients (7%) were hospitalized for the first time because of heart failure. In the latter, the adjusted risk of hospitalization for those patients with left ventricular hypertrophy (LVH) carrying the A allele was 5.5-fold greater than the G homozygous genotype. Therefore, the findings of this study propose the *KLF15* SNV rs9838915 A allele as a marker of left ventricle hypertrophy in patients with type 2 diabetes [31].

### 3.3. Krüppel-like Factors in Stroke

Stroke is a serious life-threatening condition involving the occlusion of blood supply to the brain or tissue, which can result from either the clogging (ischemic) or rupture (hemorrhagic) of an afferent artery [83,84]. In the brain, KLF-4 is a known vasculature protector, thereby acting as a guard for cerebral stroke alongside cellular adhesion molecules, such as ICAM-1 and VCAM-1. Interestingly, it could be inferred that the elevation of these molecules could serve as biomarkers to predict early stages of acute ischemic stroke [85]. Several reports on KLFs inflammatory regulatory functions have demonstrated brain transcriptional regulation of *KLF-4*, *KLF-5*, and *KLF-6* on reactive astrogliosis-derived cytokine release after ischemia [83]. Expression of *KLF-4* and the lncRNA metastasis-associated lung adenocarcinoma transcript 1 (*MALAT1)*, were significantly increased in cerebral microvascular endothelial cells after focal cerebral ischemia in cultured bEnd3 cells after an oxygen-glucose deprivation (OGD) essay that lasted for 24 h. Findings unveil that *MALAT1* targets *KLF-4* to protect the cerebral MECs after an ischemic stroke [84].

Another source of potential cerebral stroke comes from cerebral cavernous malformations (CCM) or cavernous angiomas, these malformations can lead to a low-flow hemorrhagic vascular lesion. Research on CCMs have shown that the loss-of-function of three genes is involved in the inheritance of this disease: *CCM1 (KRIT1)*, *CCM2 (Malcaverin*, *Osm)*, and *CCM3 (PDCD10)*. All of which participate in the development of nonhomologous cytoplasmic proteins of the signaling complex [86,87]. The CCM2 paralog, known as *Ccml2-like*, is a gen largely expressed in endothelial cells that has been shown to aggravate the CCM lesion in mice due to an increased expression of Map3k3-KLF signaling, particularly KLF-2 and KLF-4 [88]. The mutation leads to the activation of MEKK3 kinase cascade, which is an underlying cause of CCM lesion development and the expression of *KLF-2*, *KLF-4*, and *Adam4/5* genes [89]. Additionally, shown to be tangled in deteriorating outcomes related to stroke are mutations of the *Klf-11* gene, as certain mutations have resulted in increased blood–brain barrier permeability/leakage, an increase in the amount of water, aggravated neurobehavior performance, and reduced flow perfusion after a middle cerebral artery occlusion (MACO) in mice [83].

Blood outgrowth endothelial cells generated from strokes in children with sickle cell anemia have been found to have reduced levels of *KLF-2*. Altering the balance between proinflammatory NF-B/p65 and anti-inflammatory KLF-2, results in a proinflammatory phenotype and raises the risk of stroke in sickle cell anemia-diagnosed children [83].

### 3.4. Krüppel-like Factors in Peripheral Artery Diseases

Peripheral artery disease (PAD) is the narrowing of the vessels that carry blood (atherosclerosis) as a function of the accumulation of plaques (fat and cholesterol) in the arteries, typically but not exclusively, of the lower extremities. In recent years, researchers have uncovered the involvement of microRNAs in PAD, as microRNAs are known regulators of angiogenesis, endothelial function, inflammation, vascular regeneration, VSMCs function, restenosis, and mitochondrial function [90]. As an example, there is an intimate relation between miR-146a and KLF-4 as they regulate each other as feedback loop systems; when mir-164a targets *KLF*, VSMCs proliferation is inhibited. Nevertheless, when KLF-4 binds to miR-146a, the promoter of the miR-146a transcription is stopped, while activating *KLF-5* transcription [91]. Another important relation is seen by miR-92a, as it prevents the activation of the TNFα-mediated endothelial inflammatory response by increasing the levels of *KLF-4* [90]. During vascular injuries, miR-145 is downregulated, leading to *KLF-5* expression. Furthermore, the cluster miR-143/-145 acts as a mediator of smooth muscle cell proliferation, and a differentiator by working as a phenotypic switcher that collaborates in a network that includes KLF-4, KLF-5, and myocardin, converting enzymes, kinases, and myocardin-related factors [92]. KLF-5 is directly involved in the vascular network formation. For as seen by the overexpression of the miR-375, which directly targets to KLF-5 through regulatory changes in the NF-kB signaling pathways, a pro-angiogenic phenotype appears in endothelial cells [93].

Current strategies for patients with permanent end-organ damage related to PAD atheromatosis or with extensive aneurysmal symptomatology have focused on regulating *KLF-4* expression, as the serum levels increase as a direct impact of the endothelial, smooth muscle, and immune cells. Atherosclerosis and decreased levels of KLF-4 in the aorta are typical symptomatology of diabetes, as seen in mice models, which ultimately leads to coronary atherosclerotic heart disease, stroke, and PAD. When *KLF-4* is overexpressed in the human monocyte leukemia THP-1 cells, the cholesterol cellular levels are decreased, and it promotes the autophagy of THP-1 cells under hyper glucose conditions by the inhibition of AKT/mTOR signaling pathway, which lowers the chance to develop arthrosclerosis due to diabetic conditions [94].

### 3.5. Krüppel-like Factors in Deep Vein Thrombosis

Deep vein thrombosis (DVT) is a condition in which a clot obstructs blood flow, predominantly in the legs, and may require blood tinners, thrombolytics, support stockings, and in major cases, surgical care (thrombectomy) [95]. As earlier mentioned KLF proteins are known for networking to activate a proinflammatory phenotype in vascular structures. Transcriptome analysis has revealed that *Mmp12* and *Mmp13* can modulate *Klf-15* and *Klf-15*-related genes during a deep venous thrombosis model in C57BL/6 mice. Thus, they are aimed as potential targets for further treatments to DVT [96]. In a thrombin-induced human umbilical vein endothelial cell (HUVEC) injury model, KLF-15, and endothelial nitric oxide synthase (Enos) were shown to be upregulated and downregulated, respectively. These findings resulted in thrombin formation due to inhibition of the antithrombotic effects of Enos by *Klf-15* [95]. KLF-11 acts as main controller of the VSMCs procoagulant activity by binding to F3 to inhibit, which participates in the activation of the thrombotic phenotype. When *KLF-11* is downregulated, the extrinsic tissue factor pathway is upregulated, which leads to thrombus formation [97]. KLF-11 inhibits tissue factor gene transcription by inhibiting the expression of *EGR1* in endothelial cells. Thus, protecting against venous thrombosis [98].

### 3.6. Krüppel-like Factors in Congenital Heart Diseases

While there is still much to be addressed in developmental biology, recent research has linked KLFs to birth defects, and, in particular, certain congenital heart diseases. Out of these congenital heart diseases, the most prominently described disease linked to KLFs is the Holt–Oram syndrome. The Holt–Oram syndrome is an autosomal dominant disease characterized by upper-limb defects, congenital heart malformations, and cardiac electrical conduction-related issues. Holt–Oram syndrome has been typically associated with mutations in *TBX5*, even though new evidence has shown that KLF-13 plays a pathogenic role, as researchers have identified *KLF-13* as a genetic modifier for TBX5 [99,100]. During development, these two genes co-express in myocardium of the atrio-ventricular cushion, atrial septum, interventricular septum, and ventricular trabeculae as early as E11.5 in the mouse embryo [100]. In silico sequence analysis has further revealed conservation of binding sites on the *Nppa* promoter for both *TBX5* and *KLF-13* genes and other key cardiac transcription factors such as *Nppb*, *Vegfa*, and *Nos3*, all of which are essential for heart development. To test the existence of a genetic interaction between these two transcription factors in heart morphogenesis, Darwich et al. (2017) created a *Tbx5* and *Klf13* double heterozygote mouse model, finding significantly lower left ventricular mass over body weight ratios and atrial septal defects in 80% of the mice. Gene expression patterns of heart development regulators (*Gata4*, *Mef2a*, *Erbb4*, *Vegfc*, and *Myh7*, among others) were further analyzed in physiologically normal *Klf13* or *Tbx5* heterozygotes, as well as the double heterozygous. Their results have indicated upregulation in *Tbx5* or *Klf13* heterozygotes, yet similar to control levels in the double heterozygote mice. These findings suggest a compensatory effect between the loss of either *Klf13* or *Tbx5*, but the inability to activate these compensatory pathways when the simultaneous decrease in both transcription factors [100].

Li et al. (2020) identified two *KLF-13* variants in congenital heart disease (CHD) patients. They first identified a proline into serine transversion at the amino acid position 163 (S156N) in a patient with tricuspid valve atresia, ventricular septal defects, and atrial septal defects. Followed by the transversion of serine into asparagine at the position 156 (P163S) in a transposition of the great arteries of a seven-month patient. Both S156N and P163S were found in the nuclear localization signal 1 (NLS1) region, near to the KLF-13 DNA-binding domain. The expression of the S156N variant was noticeably higher than that of the wild-type and had increased transcriptional activity and activated the *BNP* promoter, suggesting that S156N is a gain-of-function mutation. Otherwise, the P163S variant demonstrated a similar expression compared to the wild-type yet demonstrated lower transcriptional activity. Physical interaction with TBX5 was also assessed by co-immunoprecipitation. In this concern, P163S showed a decreased physical interaction with the TBX5 protein. In contrast, S156N had a significantly increased ability to interact with TBX5. Although, authors suggest that the overexpression of KLF-13 associated with S156N might be accompanied by higher protein instability, resulting in a loss-of function phenotype [101].

Lavallée et al. have previously described similar results, identifying *Klf-13* as a modifier of *Gata4*, a key transcription factor for the cardiac natriuretic peptide genes *Nppa* and *Nppb*. In this study, *Klf-13* knockdown resulted in atrial septal defects, hypotrabeculation, and hypoplastic myocardium in Xenopus embryos [36]. Mutations in *GATA4* have previously been reported in patients with Fallot tetralogy, although no direct relation between KLF-13 and this condition has been established yet [102].

Moreover, KLF-13 seems to interact physically and functionally with GATA-6, a transcription factor expressed in smooth muscle cells and cardiomyocytes [36]. Wang et al. (2020) reported a novel *KLF-13* loss-of-function variation, with a reduced activation of *GATA-6*, *GATA-4*, and *ANP* promoters. Researchers identified this mutation in a three generation Chinese family, in which 5 out of the 18 living family members had double-outlet right ventricle and ventricular septal defects [103]. Other variations in KLF-13 have been linked to congenital heart defects, such as the Glu144*-mutant of KLF13, which cannot trans-activate *VEGF-a* and *ANP* gene promoters, which is associated with patent ductus arteriosus, ventricular septal defect, and bicuspid aortic valve [104].

Recently, *KLF-4* has been linked to Marfan syndrome, a commonly inherited connective tissue disorder caused by mutations in the *Fibrillin-1* gene, characterized by physical features such as increased height, scoliosis, arachnodactyly, lens dislocation, and cardiovascular disorders, including mitral valve prolapse and aortic aneurism, which can trigger aortic dissection [105,106]. Using single-cell sequencing, Pedroza et al. identified *KLF-4* as one of several enriched expression genes in smooth muscle cells undergoing a phenotypic modulation toward fibroblasts, in aortic aneurysm tissue from a Fbn1^C1041G/+^ Marfan syndrome murine model [107]. As we have mentioned, the regulatory effects of KLF can play a role in CVDs, and the most important mechanisms can be seen in Table 2.

**Table 2:** Krüppel -like factors involvement in different CVDs, including the mechanism affected.

Disease	KLF Involved	Effect	Mechanism	Reference
Atherosclerosis	KLF-5	Promoter	VSMCs proliferative phenotype switch via Myod repression.	[108]
	KLF-2	Protector	Reduces inflammation as it downregulates VCAM1 and E-selectin.	[14,61]
	KLF-4	Protector	Inhibition of neointima formation via SM-22 and α-SMA repression.	[109,110]
Myocardial infarction	KLF-4	Promoter	Myofibroblasts differentiation and collagen secretion via TGF-β1/Smad3 pathway.	[77]
Left ventricle hypertrophy	KLF-15	Promoter	Rs9838915 associated with increased left ventricle mass index and septal wall thickness.	[31]
Dilated cardiomyopathy	KLF-5	Promoter	Upregulation of FOXO1.	[15,34]
Diabetic cardiomyopathy	KLF-5	Promoter	Upregulation of NOX4, O−2, and ceramide accumulation.	[15]
Stroke	KLF-4	Protector	Acts as a guard by upregulating adhesion molecules ICAM-1 and VCAM-1 in the brain.	[93]
	KLF-11	Mutations	Leads to blood–brain barrier permeability,	[91]
	KLF-2	Reduction	Higher expression of proinflammatory NF-B/p65.	[91]
Peripheral artery disease	KLF-5	Maintenance	Vasculature network by collaborating with KLF-4, as myocardin, converting enzymes, kinases, and myocardin-related factors.	[102]
	KLF-4	Inhibit	Cholesterol levels reduced. Potential marker for diabetes.	[104]
Deep vein thrombosis	KLF-15	Promoter	Antithrombotic effect by upregulating nitic oxide synthetase.	[105]
	KLF-11	Reduction	Inhibits the expression of EGR1 in endothelial cells.	[107]
Congenital heart disease	KLF-13	Protection	Modifier of TBX5, protects against cardiac malformation regulating *Gata4*, *Mef2a*, *Erbb4*, *Vegfc*, and *Myh7*.	[109]

## 4. Krüppel-like Factors and miRNA in Cardiovascular Diseases

MicroRNAs (miR) are small non-coding RNAs that regulate gene expression by binding to specific messenger RNAs and either inhibiting their translation or promoting their degradation [41]. Recent studies have found that miRNAs can modulate the expression of KLFs in the context of CVDs. MicroRNA (miR)-145 is the most abundant miR in VSMC, overseeing the maintenance of cells in their contractile phenotype by promoting contractile genes [111]. The phenotype that cells are is an important factor to take into account in the pathogenesis of atherosclerosis [112]. The VSMC phenotype increases atherosclerotic development because of its facility to migrate, proliferate, and generate extracellular matrix proteins [113]. This phenotype switching is regulated by *KLF-4*, as suggested in several studies [108,114]. The overexpression of KLF-4 inhibits VSMC proliferation induced by PDGF [67,115]. miR-145 also has a role as a key regulator of *KLF-5*, *KLF-4*, and *MYOCD*, as it downregulates the first two genes by suppressing their transcription (which are repressors of MYOCD) and directly stimulates the translation of myocardin [114]. Researchers have found that miR-145 expression (KLF-5 is its target) was found to be considerably higher in the normal aortic samples group, accompanied by a higher expression of contractile proteins such as calponin and α-SMA, compared to the atherosclerotic group, where the circulating levels of miR-145 were reduced [108,116]. In animal models of vascular diseases, miR-143/miR-145 were found to be downregulated, albeit this has not been confirmed in humans. Current research further proposes that miR-145 or miR-143 are part of the regulatory loop for KLF-4, KLF-5, MYOCD, and SRF; critical transcription factors the development of SMC phenotype, and lacking SMC correct differentiation could lead more easily to the development of atherosclerosis [117,118]. *KLF-5* inhibition via miR-145 results in the failure to repress MYOCD, a transcriptional cofactor for SRF, which commands the expression of multiple smooth and cardiac muscle-specific genes, such *SM-22*, *ANP*, *MLC-2V*, and *α-MHC* [116,119,120]. The transient decease in miR-145 expression 3 days post-myocardial infarction was associated with an increase in *KLF-5* and a decrease in *MYOCD*. In addition, miR-145 is necessary for the myocardin-induced cell reprograming of adult fibroblasts into SMC and to induce the differentiation of multipotent neural crest stem cells into VSMC [121]. These data suggest that the miR-145/KLF-5/MYOCD path might be a critical modulator of VSMC in atherosclerosis.

A study using human coronary artery smooth muscle cells (HCASMCs) cultured under hyperglycemic conditions found that the repression of miR-145 resulted in *KLF-4* upregulation and thus, a decrease in MYOCD expression. This response, mediated by Ang II secretion in HCASMCs, resulted as a reaction to high glucose conditions, which developed in the facilitating migration of VSMC, as well as reduced the expression of VSMC differentiation marker genes, such as *α-SMA*, *transgelin*, and *smoothelin*, among others [114].

MiR-133 has also been associated with VSMC phenotypic modulation. miR-133 is capable of downregulating *KLF-4* via the suppression of its coactivator transcription factor, Sp1. In this process, miR-133 targets Sp-1, preventing KLF-4 activation, and making it unable to displace MYOCD from the SRF complex, determining the upregulation of smooth muscle genes, such as *MYH11* [122]. 

Horie et al. assessed the role of miR-133 in chronic heart failure, identifying *KLF-15* as another miR-133 target. In their study, miR-133 was shown to reduce KLF-15 and GLUT4 protein expression [123]. KLF-15 and MEF2A synergistically bind to the GLUT4 promoter, therefore increasing the glucose uptake in cardiomyocytes, a process of vital importance for the maintenance of myocardial energetic supply [35,124]. These results suggest that miR-133 may play a role in the perturbed energetics of heart failure. 

In another study, rats were infarcted to assess the role of miR-92a and its relation to *Klf-2* and *Klf-4* in endothelial injury after left coronary artery ligation. Herein, this study demonstrated that in animal models, endothelial injury markers, *H-Fabp*, *vWF*, and miR-92a, were significantly higher than the control group, while vasoprotective factors, *Klf-2* and *Klf-4*, were downregulated through miR-92a binding to their 3′ UTR. The suppression of miR-92a seems to promote endothelial activation, cardiac cell proliferation, and the decrease in apoptosis after AMI, proving that both *Klf-2* and *Klf-4* are involved in the protection and modulation of endothelial cells [125]. Similar results were obtained when using antimiR-92a, decreased macrophage and T lymphocyte accumulation, and a marked reduction in atherosclerosis (32%, as compared to the non-treated group) [126]. 

miR-32-5p targets the expression of *KLF-2*. In a recent study, researchers found elevated serum levels of miR-32-5p in patients with AMI, and reduced expressions of KLF-2 [127]. KLF-2 possesses atheroprotective properties [125]; therefore, having an adequate expression of this gene could prevent the development of a cardiovascular disease. In another study, miR-363-3p was upregulated in the serum of AMI patients, showing that the expression of this miR was positively correlated with the concentration of endothelial injury biomarkers. As confirmed in rat studies with a knockdown of miR-363-3p, which showed that endothelial injury biomarkers are reduced. In the same study, they observed that the activity of KLF-2 was inhibited with the upregulation of miR-363-3p, leading patients toward a higher probability of suffering AMI [128].

Regarding ischemic damage, miR-125b-5p was linked to a cardioprotective effect in the onset of AMI. Using several prediction algorithms, researchers have identified proapoptotic *BAX1* and *KLF-13* as miR-125b-5p targets. In this study, this research group showed that in vivo repression of miR-125b-5p is associated with a higher mortality after left coronary artery ligation, left ventricular disfunction, enhanced susceptibility to cardiac rupture, higher levels of *ANP* and *TNF-α*, and larger fibrotic regions. An in vitro analysis showed that miR-125b-5p could induce an increase in p-AKT levels, suggesting a function as a pro-survival miR in cardiomyocytes. Furthermore, researcher proved that β-blocker carvedilol was capable of upregulating miR-125b-5p (a process accompanied by a decrease in BAX1 and KLF-13) [129]. These findings suggest miR-125b-5p to be a carvedilol-responsive miR, a mediator of improved cardiac function after AMI, via the blockage of pro-apoptotic proteins.

miR-let-7g demonstrated an increase in the expression of α-SMA and calponin by the downregulation of PDGF-β, leading to a reduced interaction between KLF-4 and SRF, which de-repressed MYOCD; this maintains VSMC contractile phenotype and therefore reduced the formation of atherosclerotic plaques [130]. There have been some miRNAs that are related with the AMI but not associated with KLF signaling. miR-139-5p has been involved in regulating cardiomyocyte proliferation and apoptosis. Finally, further research has also confirmed that miR-139-5p increases in the serum of AMI patients [131]. An overview of miRs involved in the regulation of CVDs, as well as their target and response can be seen on Table 3.

**Table 3:** miRNAs involvement in KLF regulation during CVDs.

MiRNAs	Cardiovascular Diseases	Target	Response	Ref.
miR-143/145 miR-1 miR-137-3p	Promotes atherosclerosis	KLF-4/5 KLF-4 KLF-15	↓ Expression; MiR-1 induces SMC differentiation through the repression of Klf4; Promote ischemia.	[132,133,134]
miR126	Promotes atherosclerosis	KLF-2	↑ expression of miR-126 ↑ KLF-2 activated VEGF.	[135]
miR29a	Promotes atherosclerosis	KLF-15	↑ expression of miR-29a ↑ miR29 increased KLF-15 stability by Fbw7/CDC4.	[136]
miR-410 mmu-miR-107, mmu-miR-142-5p, mmu-miR-143, mmu-miR-155	Anti-atherosclerosis	KLF-5 KLF-2	HDAC1; KLF-5 promotes IKB alpha ↓ NFκB; FOXO1 regulates the expression of the downstream transcription factor KLF-2 in endothelial cells.	[137,138]
miR-10a	Myocardial infarction	KLF-4	miR-10a rejuvenated aged hBM-MSCs, which improved angiogenesis and cardiac function in injured mouse hearts.	[139]
miR-27a	Myocardial infarction	KLF-5	miR-27a expression could be transcriptionally suppressed by KLF-5 and inactivated by the TGF-β/Smad2/3 signaling pathway.	[140]
mIR-363-3p	Myocardial infarction	KLF-2	↓ miR-363-3p reduces the concentration of endothelial biomarkers and promotes the vascular endothelial cell proliferation, and this protective effect on endothelial injury may be exerted by targeting KLF2.	[128]
miR32-5p	Myocardial infarction	KLF-2	miR-32-5p promotes endothelial cell viability.	[127]
miR-125b-5p	Myocardial infarction	KLF-13	miR-125b-5p protects the heart against AMI by blunting CM death in response to injury in part through its repression of bak1 and klf13.	[129]
miR-150	Myocardial infarction	KLF-13	Increasing KLF-13 expression via ↓ miR-150.	[141]
mIR-92a	Myocardial infarction	KLF-2 KLF-4	miR-92a promoted endothelial activation, cardiac cell proliferation. and apoptosis decrease after AMI, proving that both KLF-2 and KLF-4 are involved in the protection and modulation of endothelial cells.	[142]
miR-124	Atherosclerosis	KLF-6 and STAT3	Downregulation of miR-124 and Sp1 levels was found in human aortic media from clinical specimens of aortic dissection.	[143,144]
miR-let-7g	Atherosclerosis	KLF-4, SRF, α-SMA, calponin, and PDGF-B	↑ α-SMA expression via ↓ KLF-4 & SRF which depresses Myod.	[130]

## 5. Conclusions

The extensive KLFs family has been associated with many biological processes that are related to cell growth, differentiation, and death, and the development and maintenance of tissues in many eukaryotic organisms. In cardiovascular system dysregulation, KLFs seem to be associated with CVDs, such as (a) CHD-linked syndromes or malformations because of autosomal diseases related to instability and/or loss of function, (b) arterial damage leading to stroke, (c) ischemic damages due to differentiation of cardiac myofibroblasts or a modified fatty acid oxidation as related to the formation of a dilated cardiomyopathy, and (d) cardiovascular complications such as myocardial infarctions, left ventricular hypertrophy, and diabetic cardiomyopathies. Finally, several miR have been linked to AMI, but not all are related to KLFs signaling. Others miR have been involved in certain regulatory loops of KLFs as they may act as critical modulators of VSMC in atherosclerosis, abnormalities of heart failure, and as markers of endothelial damage in the AMI.

## Data Availability

Not applicable.
